# Common polymorphic inversions at 17q21.31 and 8p23.1 associate with cancer prognosis

**DOI:** 10.1186/s40246-019-0242-2

**Published:** 2019-11-21

**Authors:** Carlos Ruiz-Arenas, Alejandro Cáceres, Victor Moreno, Juan R. González

**Affiliations:** 1 Barcelona Institute for Global Health, ISGlobal, Doctor Aiguader 88, 08003 Barcelona, Spain; 2 Universitat Pompeu Fabra (UPF), Barcelona, Spain; 3 CIBER Epidemiología y Salud Pública (CIBERESP), Barcelona, Spain; 4 Programa de Prevención y Control del Cáncer, Instituto Catalán de Oncología, L’Hospitalet, Barcelona, Spain

**Keywords:** Chromosomal inversions, Cancer prognosis, DNA methylation, Genetic epidemiology, Gene expression

## Abstract

**Background:**

Chromosomal inversions are structural genetic variants where a chromosome segment changes its orientation. While sporadic de novo inversions are known genetic risk factors for cancer susceptibility, it is unknown if common polymorphic inversions are also associated with the prognosis of common tumors, as they have been linked to other complex diseases. We studied the association of two well-characterized human inversions at 17q21.31 and 8p23.1 with the prognosis of lung, liver, breast, colorectal, and stomach cancers.

**Results:**

Using data from The Cancer Genome Atlas (TCGA), we observed that inv8p23.1 was associated with overall survival in breast cancer and that inv17q21.31 was associated with overall survival in stomach cancer. In the meta-analysis of two independent studies, inv17q21.31 heterozygosity was significantly associated with colorectal disease-free survival. We found that the association was mediated by the de-methylation of cg08283464 and cg03999934, also linked to lower disease-free survival.

**Conclusions:**

Our results suggest that chromosomal inversions are important genetic factors of tumor prognosis, likely affecting changes in methylation patterns.

## Introduction

Chromosomal inversions are structural genetic variants where a chromosome segment changes its orientation with respect to a reference genome. Chromosomal inversions are either sporadic or polymorphic. Sporadic inversions are infrequent new mutations that have been linked to cancer susceptibility [1–3] and progression [4]. For instance, a sporadic inversion in chromosome 16 is a known precursor of leukemia (reviewed in [5]). By contrast, polymorphic inversions are common variants in the population. Ancient non-recurrent inversions define divergent haplotypes, each linked to an inversion status, as inverted and standard chromosomes do not recombine [6]. Based on this observation, different methods on nucleotide variation data have been implemented to call inversions status from haplotype differences [7, 8]. Thus, the re-analysis of existing GWAS data and bioinformatics tools have allowed the study of the role of polymorphic inversions in complex diseases, such as asthma and obesity [9], neuroticism [10], and ovarian cancer [11]. Since no study has reported associations with cancer prognosis, we asked the extent to which polymorphic inversions are also related to the prognosis of common cancers that included lung, liver, stomach, breast, and colorectal.

We studied the role of the inversions at 8p23.1 and 17q21.31 in cancer prognosis as these two inversions are well-characterized and can be genotyped to high accuracy using SNP array data [6, 8, 12]. Gene expression and methylation data analyses were performed to assess the transcriptomic and epigenomic effects of inversions and their potential effects on prognosis. Mediation analyses were carried out to determine whether gene expression or DNA methylation are suitable mediators of the association between inversions and cancer prognosis.

## Materials and methods

### Inversion calling on TCGA

We obtained TCGA SNP data in Birdseed format from NCI Genomic Data Commons (GDC) legacy archive [13]. We converted the data to VCF format and mapped them to the human assembly hg19 using birdseed2vcf [14]. We imputed the SNPs with the Michigan server [15], using HRC Version r1.1 2016 as the reference and SHAPEIT v2.r790 as the phasing algorithm. We used peddy [16] to select individuals detected as European with a confidence higher than 0.9. Inversion genotypes for inv8p23.1 and inv17q21.31 were obtained using *scoreInvHap* that uses SNP information on inversion regions to call inversion genotypes [8, 17].

### CRCGEN

The CRCGEN study combines data of three case-control studies performed in Spain. The first study was performed in the University Hospital of Bellvitge, L’Hospitalet, Barcelona, and recruited 304 incidents, pathology confirmed, colorectal cancer (CRC) cases and 293 age and sex frequency-matched hospital controls during the period 1996–1998. The second study, performed in the same hospital during the period 2007–2015, included a total of 324 cases and 376 population controls. The third study was conducted in Hospital of León, León, during 2008–2013. A total of 325 incident CRC cases and 407 population controls were included. Written informed consent was required from all participants. Each Hospital’s ethics committees (Bellvitge and León) approved the protocols of the study. The three studies contributed to CORECT consortium, so genotyping and quality control was performed simultaneously for all subjects.

### Survival analysis

We selected the cancers with the highest worldwide mortality [18]: lung, liver, colorectal, stomach, and breast. In TCGA, these cancers corresponded to LUAD (lung adenocarcinoma), LUSC (lung squamous cell carcinoma), LIHC (liver hepatocellular carcinoma), COAD (colon adenocarcinoma), READ (rectum adenocarcinoma), STAD (stomach adenocarcinoma), and BRCA (breast invasive carcinoma). We considered LUAD and LUSC as two independent cancers and COAD and READ as one single cancer (i.e., colorectal). We only considered female samples for breast cancer associations. We downloaded TCGA clinical data using *curatedTCGAData* [19]. We fitted survival and disease-free-survival (i.e., recurrence) Cox proportional hazards models. Inversion genotypes for inv17q21.31 and inv8p23.1 were considered as risk factors under four different genetic models: (1) additive (Std-Std, 0; Std-Inv, 1; Inv-Inv, 2); (2) dominant (Std-Std, 0; Std-Inv, 1; Inv-Inv, 1); (3) recessive (Std-Std, 0; Std-Inv, 0; Inv-Inv, 1), and (4) overdominant (Std-Std, 0; Std-Inv, 1, Inv-Inv; 0). We accounted for multiple testing using Bonferroni correcting for four genetic models, considering significant *p* values that were lower than 1.19 × 10^−3^. For all tumors, we tested a univariate and a multivariate model adjusted for age, gender, pathologic stage (stage I, stage II, stage III, and stage IV), and the first four genome-wide principal components inferred by peddy [16].

Using the CRCGEN study, we tested the replication of the significant associations found for colorectal cancer. We genotyped inversions using *scoreInvHap* on 760 patients with complete information on the selected covariates. We fitted a frailty Cox proportional hazard model for the significant associations previously found, adjusting for age, gender, pathologic stage, cancer site, and recruitment city as random effect to control for possible confounding related to recruiting process. The asymptotic power based on an approximate variance formula implemented in the survSNP R package [20] was used to estimate the power of replicating the increased risk of colorectal recurrence and inversion 17q21.31 assuming an additive model (overdominant is not implemented in the package). We meta-analyzed the results of TCGA and CRCGEN models using *metafor* R package [21].

### Gene expression analysis

We downloaded the GDC harmonized version of gene expression data using *TCGAbiolinks* [22]. We merged COAD and READ datasets and we selected samples from primary tumor, with reported pathologic stage and with inversion status inferred by *scoreInvHap*. We removed genes with less than ten counts in more than 1% of the samples and we transformed count values to log_2_ CPMs using *voom* [23]. The final dataset contained 477 individuals and 27,291 genes, where we tested the association between gene expression and inv17q21.31 using robust linear models and redundancy analysis (RDA) [24], as implemented in *MEAL* [25]. Both models included age, gender, pathologic stage, PC genetic components, and 53 surrogate variables as covariates. We accounted for multiple testing in robust linear model analysis using Benjamini-Hochberg method [26]. The results were mapped to gene coordinates in human assembly hg19 using *biomaRt* [27, 28].

### DNA methylation analysis

We downloaded the GDC harmonized version of DNA methylation data using *TCGAbiolinks*. We merged COAD and READ datasets and we selected samples from primary tumor. We removed probes with SNPs as defined in the *minfi* package [29], in sexual chromosomes and likely to cross-hybridize [30]. The final dataset contained 265 individuals and 350,879 CpGs. *MEAL* package [25] was used to associate inv17q21.31 with DNA methylation. We fitted robust linear models to detect differentially methylated probes (DMP); we also used redundancy analysis in the inverted region and three methods to detect differentially methylated regions (DMRs): bumphunter [31], blockFinder [29], and DMRcate [32]. All the models included age, gender, pathologic stage, PC genetic components, and 37 surrogate variables as covariates. We accounted for multiple testing in robust linear model analysis using Benjamini-Hochberg adjustment. We reported the genes mapped to CpG using Release 93 of ENSEMBLE nomenclature.

### Mediation analysis

We evaluated whether gene expression or DNA methylation were mediators of the association between inversion inv17q21.31 and colorectal recurrence. We accounted for technical bias on gene expression and DNA methylation by computing residuals, removed from the effect of surrogate variables. We evaluated whether gene expression mediated the effect of inv17q21.31 on tumor recurrence using the genes previously associated with the inversion. Four hundred seventy-seven samples were available with gene expression and clinical data. The mediation test included a generalized linear model (gene vs inversion) and a regression parametric model (tumor recurrence vs inversion + gene), both adjusted for age, sex, pathologic stage, and the first four genome-wide principal components. We run 1000 permutations to compute the significance of the mediation and used the same method for the mediation of the association between inv17q21.31 and disease-free survival. We tested whether the CpGs affected by the inversion associated with tumor recurrence, using a Cox proportion hazards regression model. We selected those CpGs associated with tumor recurrence either in a crude model or after adjusting for age, sex, pathologic stage, and the first four genome-wide principal components (*p* value < 0.05). We performed mediation tests with the *mediation* R package [33].

## Results

### Chromosomal inversions associate with overall and disease-free cancer survival

Table 1 shows the patients characteristics included in the study. We did not find an association between chromosomal inversions at 8p23.1 and 17q21.31 and general patients’ features.

**Table 1:** Individual characteristics in TCGA datasets

	Lung1 (*n* = 381)	Lung2 (*n* = 399)	Liver (*n* = 140)	Colorectal (*n* = 470)	Stomach (*n* = 240)	Breast (*n* = 734)
Inv8p23.1						
Std-Std	59 (15.5%)	81 (20.3%)	21 (15.0%)	88 (18.7%)	55 (22.9%)	128 (17.5%)
Std-Inv	205 (53.8%)	207 (51.9%)	83 (59.3%)	219 (46.6%)	115 (47.9%)	376 (51.2%)
Inv-Inv	117 (30.7%)	111 (27.8%)	36 (25.7%)	163 (34.7%)	14 (29.2%)	230 (31.3%)
Inv17q21.31						
Std-Std	225 (59.1%)	244 (61.1%)	83 (59.3%)	294 (62.7%)	158 (65.8%)	453 (61.7%)
Std-Inv	140 (36.7%)	128 (32.1%)	49 (35.0%)	162 (34.5%)	68 (28.3%)	250 (34.1%)
Inv-Inv	16 (4.2%)	27 (6.8%)	8 (5.7%)	14 (2.97%)	14 (5.8%)	31 (4.2%)
Age (years)	67 (33-88)	69 (40-90)	65 (17-85)	69 (31-90)	67 (41-90)	60 (26-90)
Sex						
Women	205 (53.8%)	99 (24.8%)	68 (48.6%)	225 (47.9%)	93 (38.8%)	734 (100%)
Men	176 (46.2%)	300 (75.2%)	72 (51.2%)	245 (52.1%)	147 (61.3%)	0 (0%)
Tumor stage						
Stage I	210 (55.1%)	198 (49.6%)	67 (47.9%)	88 (18.7%)	35 (14.6%)	129 (17.6%)
Stage II	87 (22.8%)	129 (32.3%)	36 (25.7%)	176 (37.4%)	68 (28.3%)	404 (55.0%)
Stage III	64 (16.8%)	66 (16.6%)	34 (24.3%)	138 (29.4%)	112 (46.7%)	179 (24.4%)
Stage IV	20 (5.3%)	6 (1.5%)	3 (2.1%)	68 (14.5%)	25 (10.4%)	22 (3.0%)
Follow-up time (days)	609 (0-7248)	671 (0-4765)	662 (0-3478)	648 (0-4502)	415 (0-3720)	838 (0-8605)

Continuous variables are described with median and range. Categorical variables are described with counts and the percentages of each category

*Lung1* LUAD (lung adenocarcinoma), *Lung2* LUSC (lung squamous cell carcinoma), *Liver* LIHC (liver hepatocellular carcinoma), *Colorectal* COAD + READ (colon adenocarcinoma), *Stomach* STAD (Stomach adenocarcinoma), *Breast* BRCA (breast invasive carcinoma)

We tested the association of inv8p23.1 and inv17q21.31 with overall survival using an unadjusted model (Table 2). We observed that the inverted homozygous for inv8p23.1 associated with lower breast cancer survival (HR 2.01, *p* value 2.7 × 10^−3^) but with higher stomach cancer survival (HR 0.42, *p* value 3.3 × 10^−2^), whereas standard homozygous for inv17q21.31 associated with low survival of stomach cancer (HR 2.19, *p* value 1.1 × 10^−2^). After adjusting for sex, age, tumor stage, and the first four genetic principal components, we found that the association between inv8p23.1 and breast cancer survival further increased (HR 2.55, *p* value 1.4 × 10^−4^), likewise the association between inv17q21.31 and stomach cancer survival (HR 3.26, *p* value 5.8 × 10^−4^) (Additional file 1, Supplementary Tables 1–2). However, the adjustment removed the significant association between inv8p23.1 and stomach cancer (HR 0.62, *p* value 0.14) (Additional file 1, Supplementary Table 2). Note that all reported associations were statistically significant under Bonferroni threshold (1.19 × 10^−3^). Multivariate models confirmed that pathologic stage and age are strong predictors of overall survival (Additional file 1, Supplementary Tables 1–6).

**Table 2:** Hazard ratios (HR) of overall survival using Cox regression models

Tumor	inv8p23.1				inv17q21.31			
	Std-Std	Std-Inv	Inv-Inv	*p* value	Std-Std	Std-Inv	Inv-Inv	*p* value
Lung1	1.10 (0.80-1.50)			0.55	0.78 (0.55-1.12)			0.18
Lung2	1	1	0.96 (0.65–1.42)	0.84	1	1	0.72 (0.35–1.47)	0.37
Liver	1	1	0.84 (0.45–1.55)	0.58	1	0.74 (0.42-1.30)	1	0.30
Colorectal	1	0.68 (0.38–1.19)	1	0.18	1	0.74 (0.40-1.36)	1	0.33
Stomach	*1*	*1*	*0.42 (0.18–0.93)*	*3.3 × 10*^*−2*^	*1*	*2.19 (1.20–3.99)*	*2.19 (1.20–3.99)*	*1.1 × 10*^*−2*^
Breast	*1*	*1*	*2.00 (1.27–3.16)*	*2.6 × 10*^*−3*^	1.34 (0.93–1.94)			0.12

The results are shown for the best genetic model for each inversion in each tumor. Associations in italics were nominally significant (*p* value < 0.05). In the additive model, HR corresponds to each inverted allele. For the other models, HR was computed using Std-Std as reference

*Lung1* LUAD (lung adenocarcinoma), *Lung2* LUSC (lung squamous cell carcinoma), *Liver* LIHC (liver hepatocellular carcinoma), *Colorectal* COAD + READ (colon adenocarcinoma), *Stomach* STAD (stomach adenocarcinoma), *Breast* BRCA (breast invasive carcinoma)

**Fig. 1:**
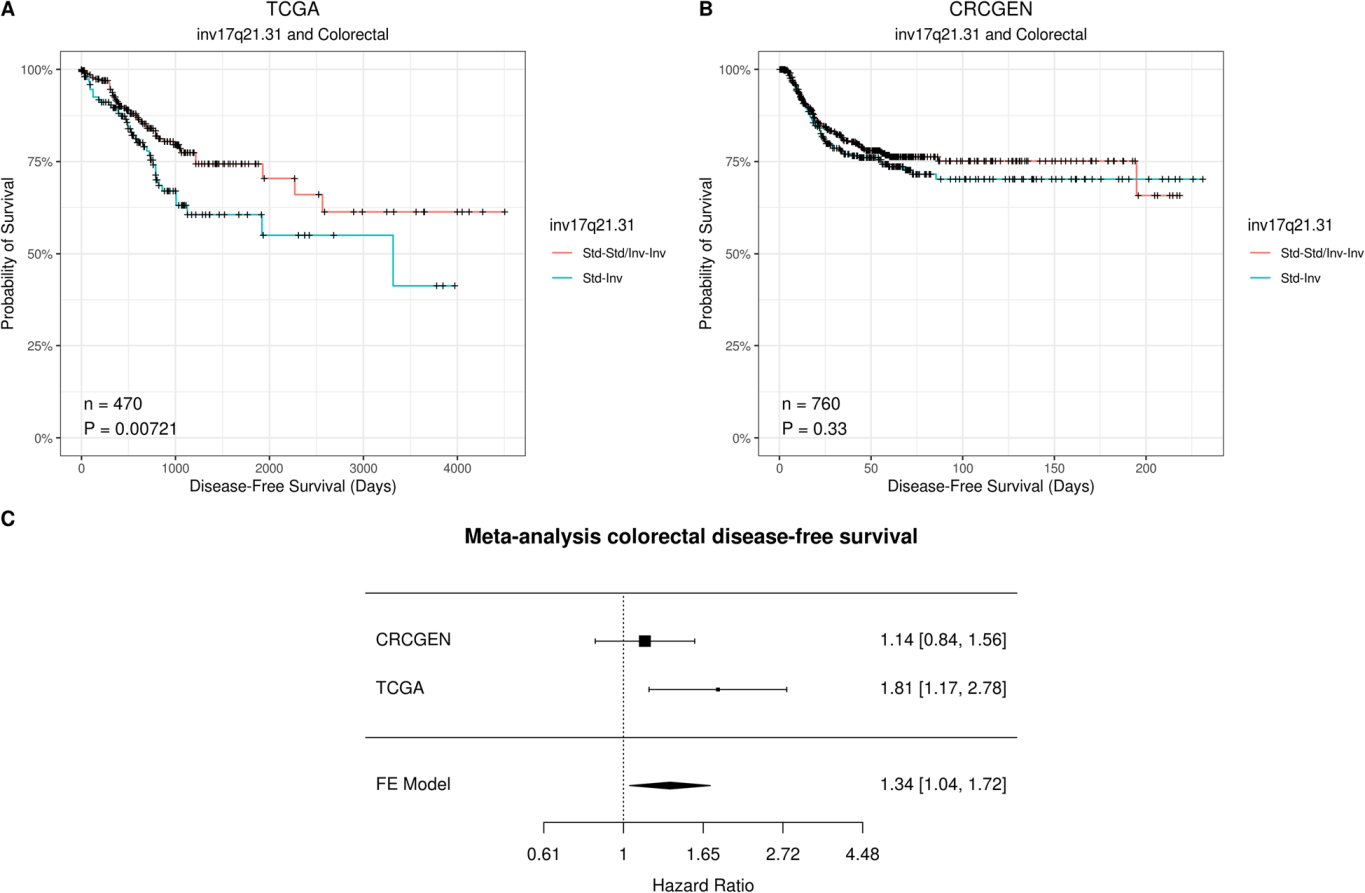
Effect of inv17q21.31 on colorectal disease-free survival. **a**, **b** Disease-free survival of colorectal cancer for inversion inv17q21.31 in TCGA (**a**) and CRCGEN (**b**) under the overdominant model. **c** Meta-analysis of TCGA and CRCGEN studies

We then tested the association between inv8p23.1 and inv17q21.31 with disease-free survival (Table 3). Only one significant association was significant, between heterozygous individuals for inv17q21.31 and decreased tumor disease-free survival in colorectal cancer (HR 1.67, *p* value 1.6 × 10^−2^) (Fig. 1, Table 3). After adjusting for age, sex, tumor stage, and the first four genetic principal components, the association was on the limit of Bonferroni correction (HR 1.81, *p* value 7.2 × 10^−3^) (Additional file 1, Supplementary Table 7). Such overdominant model is plausible as inversion heterozygous affect chromosome pairing which can lead to genomic alterations [34]. In addition, the multivariate models confirmed that the pathologic stage is a strong predictor of disease-free survival (Additional file 1, Supplementary Tables 7–12).

**Table 3:** Crude Cox regression models between chromosomal inversions and disease-free survival

Tumor	inv8p23.1				inv17q21.31			
	Std-Std	Std-Inv	Inv-Inv	*p* value	Std-Std	Std-Inv	Inv-Inv	*p* value
Lung1	0.78 (0.61–1)			0.05	0.88 (0.67–1.16)			0.37
Lung2	1	0.82 (0.52–1.28)	0.82 (0.52–1.28)	0.38	1	1	0.49 (0.2–1.2)	0.12
Liver	1	1	1.01 (0.61–1.68)	0.96	1	1.13 (0.74–1.74)	1.13 (0.74–1.74)	0.57
Colorectal	1	0.86 (0.51–1.44)	0.86 (0.51–1.44)	0.56	*1*	*1.67 (1.1–2.53)*	*1*	*1.57 × 10*^*−2*^
Stomach	1	1	0.79 (0.44–1.4)	0.42	1	0.98 (0.57–1.68)	1	0.93
Breast	1	0.66 (0.41–1.04)	1	0.08	1	1	2.01 (0.81–4.99)	0.13

The results are for the best genetic model for each inversion in each tumor. Associations in italics were nominally significant (*p* value < 0.05). In the additive model, HR corresponds to each inverted allele. For the other models, HR was computed using Std-Std as the reference

*Lung1* LUAD (lung adenocarcinoma), *Lung2* LUSC (lung squamous cell carcinoma), *Liver* LIHC (liver hepatocellular carcinoma), *Colorectal* COAD + READ (colon adenocarcinoma), *stomach*: STAD (stomach adenocarcinoma), *Breast* BRCA (breast invasive carcinoma)

We then tested the replication of inv17q21.31 association using the colorectal CRCGEN study. We had a 99.5% power to detect a HR = 1.81 for recurrence assuming *α* = 0.05, a 0.24 inversion allele frequency, 0.21 recurrent event rate, and an additive model. Participants of this study had different characteristics than TCGA patients (Additional file 1, Supplementary Table 13). We observed, in a fully adjusted model (age, sex, tumor stage, and patients’ city), that while heterozygous individuals for inv17q21.31 decreased tumor disease-free survival, the association was not statistically significant (HR 1.16, *p* value 0.33) (Additional file 1, Supplementary Table 14). However, the association was significant in the meta-analysis of TCGA and CRCGEN studies (HR 1.34, *p* value 2.3 × 10^−2^) (Fig. 1). We further asked whether the observed overdominance of inv17q21.31 in colorectal disease-free survival was supported by functional associations with gene expression and DNA methylation in the TCGA study.

### inv17q21.31 effect on colorectal disease-free survival is more likely mediated by DNA methylation than by gene expression

We aimed to find a molecular mechanism to explain the effect of inv17q21.31 on colorectal disease-free survival using TCGA data. To this end, we tested two different hypotheses: (1) a change in the expression of a gene mediates the association between the inversion and disease-free survival and (2) specific changes in DNA methylation, which may regulate the expression of several genes and mediate the association between the inversion and disease-free survival.

Heterozygous for inv17q21.31 were associated with significant differences in the expression of 12 genes within inv17q21.31 region (Additional file 1, Supplementary Table 15) and explained 10% of the gene expression variability (Additional file 1, Supplementary Figure 2). At genome-wide level, inversion inv17q21.31 changed the expression of another five genes (Additional file 1, Supplementary Table 15). However, none of the genes affected by the inversion mediated the association between inv17q21.31 and colorectal disease-free survival.

**Fig. 2:**
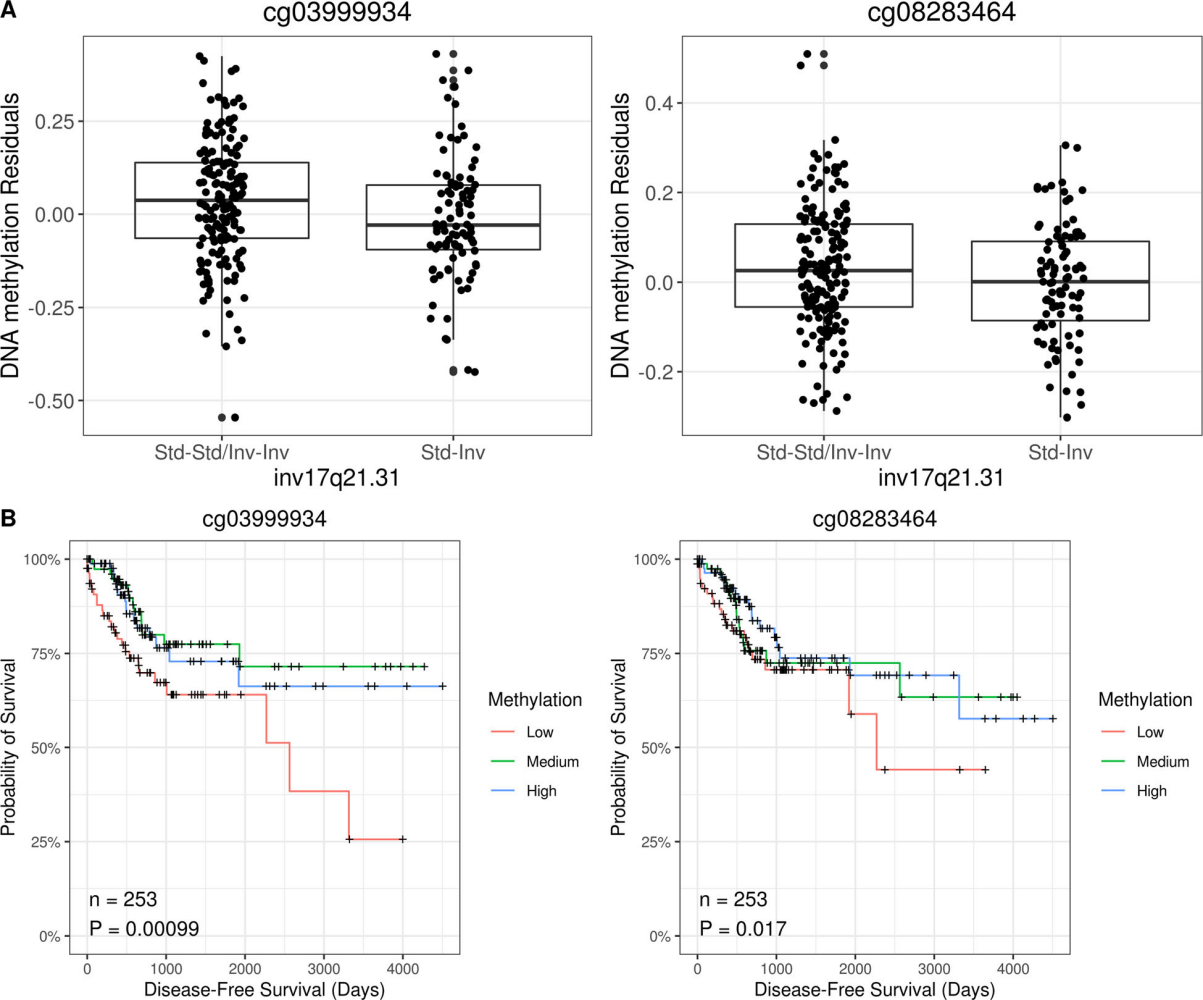
CpGs mediating the association between inversion inv17q21.31 and disease-free survival in colorectal cancer. **a** Boxplots of DNA methylation versus inversion inv17q21.31 genotypes. **b** Survival curves for each CpG and colorectal disease-free survival in TCGA. *p* values correspond to Cox proportional hazard regression where CpG is considered a continuous variable and the model is adjusted for confounders

Heterozygous for inv17q21.31 were associated with significant changes in methylation of 11 CpGs inside the inversion region (Additional file 1, Supplementary Table 16). However, the CpGs only explained 1% of methylation variability (Additional file 1, Supplementary Figure 3). Significant methylated regions (DMRs) in inv17q21.31 were also detected with Bumphunter and DMRcate for inverted heterozygous (Additional file 1, Supplementary Tables 17–18). At genome-wide level, inv17q21.31 changed the methylation of other 87 CpGs in different chromosomes (Additional file 1, Supplementary Table 16). We found that six of these CpGs also associated with disease-free survival. We then tested the mediation of these six CpGs in the association between the inversion and disease-free survival and found two CpGs with significant mediation effects: cg08283464 mediated a 15.0% of the association (*p* value, 0.048) and cg03999934 a 20.7% (*p* value, 0.032). In particular, both CpGs had lower methylation in heterozygous individuals (Fig. 2a, Additional file 1, Supplementary Table 16), consistent with the observation that lower methylation values were associated to lower tumor disease-free survival (HR 0.015, *p* value 0.017 for cg08283464; HR 0.034, *p* value 9.9·10^−4^ for cg03999934) (Fig. 2b, Additional file 1, Supplementary Table 19).

## Discussion

We found that chromosomal inversions at 8p23.1 and 17q21.31 affect tumor prognosis in breast, stomach, and colorectal cancer. These new biomarkers should be further considered in prognosis assessment in addition to the SNPs associated with breast and stomach cancer survival [35–37] and with colorectal cancer recurrence [38, 39] and in addition to germline CNVs associated with breast and colorectal cancer prognosis [40–42]. As such, further studies need to evaluate the increased power of polygenic scores of prognosis and susceptibility given by the inclusion of these inversions [43]. The inversions have the potential to improve polygenic scores by including common genomic structural variants and by specifically including variants associated with prognosis [44].

Inversions inv8p23.1 and inv17q21.31 were associated with overall survival based on dominant and recessive genetic models. Both inversions have already been associated with different diseases. inv8p23.1 has been associated with system systematic lupus [45, 46], neuroticism [10], autism [47], schizophrenia [47], and underweight [12], and inv17q21.31 has been associated with Parkinson [48–51], neurodegenerative tauopathies [52, 53], Alzheimer’s disease [54], neuroticism [10], autism [47], schizophrenia [47], or response to corticosteroids in asthma [55].

Inversion heterozygous at 17q21.31 predicted lower disease-free survival in colorectal cancer. While overdominance is uncommon for SNPs, inversion heterozygous have shown deleterious effects on complex phenotypes, such as congenital ichthyosis [56], where non-allelic homologous recombination (NAHR) that reverts the effect of detrimental mutations is impaired in inverted heterozygous. A similar mechanism could explain the worse colorectal cancer prognosis of inverted heterozygous. Another mechanism for the overdominant effect of the inversion could be linked to the deletion of the region during mitosis, as inverted heterozygous favor the generation of such chromosome rearrangements [34]. Further research is needed to elucidate the specific mechanisms for the lower prognosis of inv17q21.31 heterozygous.

In this work, we tested two possible mediators between inversion inv17q21.31 and disease-free survival: (1) expression changes in specific genes and (2) DNA methylation changes in specific CpGs, which could correlate with the expression of several genes. Our results support DNA methylation changes as the more likely mediators. We did not observe a mediation effect of these genes on the overdominance of inv17q21.31 on disease-free survival, although inv17q21.31 heterozygous were associated with gene expression on colorectal tumors, in line with previous studies in blood and brain [53, 57–60]. However, we cannot discard that the overall mediatory effect is given by the additive contribution of small independent effects of each gene, for which there is lack of statistical power. On the other hand, the association between inv17q21.31 heterozygous with extensive genome-wide changes in DNA methylation on colorectal tumor tissue underlines the genome-wide role of the inversion, already observed for genome-wide gene expression changes in blood [53], and global recombination [61]. We found that the two CpGs that partially mediated the effect of inv17q21.31 on colorectal disease-free survival are intergenic and have the potential to affect the transcription of several genes. While DNA methylation clearly affects colorectal recurrence [62, 63] and changes in DNA methylation have also been observed to mediate the effect of inv17q21.31 on diseases [53], the effect of inv17q21.31 in global epigenetic patterns needs further investigation.

In conclusion, we offer novel evidence on the effect of common inversion polymorphisms on the tumor prognosis of common cancers, indicating underlying epigenomic mechanisms linking inv17q21.31 to colorectal disease-free survival. Although more research is needed to validate the associations between inv17q21.31 heterozygosity and colorectal cancer disease-free survival, we show significant functional correlations that support our observations.

## Supplementary information

**Additional file 1.** Supplementary Figures and Tables (.pdf).

## Data Availability

Data from TCGA is available from Genomic Data Commons: https://gdc.cancer.gov/. TCGA genetic data was obtained from dbGaP: accession number phs000178.v10.p8. Data from CRCGEN will be uploaded to a public repository after manuscript acceptance.
