# Supplementary material for: Common polymorphic inversions at 17q21.31 and 8p23.1 associate with cancer prognosis
Source: Hum Genomics. 2019 Nov 21;13:57. doi: 10.1186/s40246-019-0242-2 (PMC6873427; doi:10.1186/s40246-019-0242-2)
Supplement: Supplementary file 1 — Additional file 1. Supplementary Figures and Tables (.pdf). [file 40246_2019_242_MOESM1_ESM.pdf]

Supplementary figures and tables

Supplementary Figure 1: Effect of inversions on cancer prognosis. 3

Supplementary Figure 2: Redundancy analysis of inversion inv17q21.31 on gene expression in colorectal cancer. 4

Supplementary Figure 3: Redundancy analysis of inversion inv17q21.31 on DNA methylation in colorectal cancer. 5

Supplementary Table 1: Adjusted Crude Cox regression models between chromosomal inversions and overall survival in breast cancer. 6

Supplementary Table 2: Adjusted Crude Cox regression models between chromosomal inversions and overall survival in stomach cancer. 6

Supplementary Table 3: Adjusted Crude Cox regression models between chromosomal inversions and overall survival in lung adenocarcinoma. 7

Supplementary Table 4: Adjusted Crude Cox regression models between chromosomal inversions and overall survival in lung squamous cell carcinoma. 7

Supplementary Table 5: Adjusted Crude Cox regression models between chromosomal inversions and overall survival in liver cancer. 8

Supplementary Table 6: Adjusted Crude Cox regression models between chromosomal inversions and overall survival in colorectal cancer. 8

Supplementary Table 7: Adjusted Crude Cox regression models between chromosomal inversions and disease-free survival in colorectal cancer. 9

Supplementary Table 8: Adjusted Crude Cox regression models between chromosomal inversions and disease-free survival in breast cancer. 9

Supplementary Table 9: Adjusted Crude Cox regression models between chromosomal inversions and disease-free survival in stomach cancer. 10

Supplementary Table 10: Adjusted Crude Cox regression models between chromosomal inversions and disease-free survival in lung adenocarcinoma. 10

Supplementary Table 11: Adjusted Crude Cox regression models between chromosomal inversions and disease-free survival in lung squamous cell carcinoma. 11

Supplementary Table 12: Adjusted Crude Cox regression models between chromosomal inversions and disease-free survival in liver cancer. 11

Supplementary Table 13: Participant features in CRCGEN. 12

Supplementary Table 14: Cox regression models between inv17q21.31 and colorectal disease-free survival in CRCGEN. 12

Supplementary Table 15: Genes modified by inversion inv17q21.31. 13

Supplementary Table 16: CpGs affected by inversion inv17q21.31 in colorectal cancer. 14

Supplementary Table 17: DMRs detected with bumphunter 17

Supplementary Table 18: DMRs detected with DMRcate 17

Supplementary Table 19: Cox regression models between CpGs and colorectal prognosis. 17

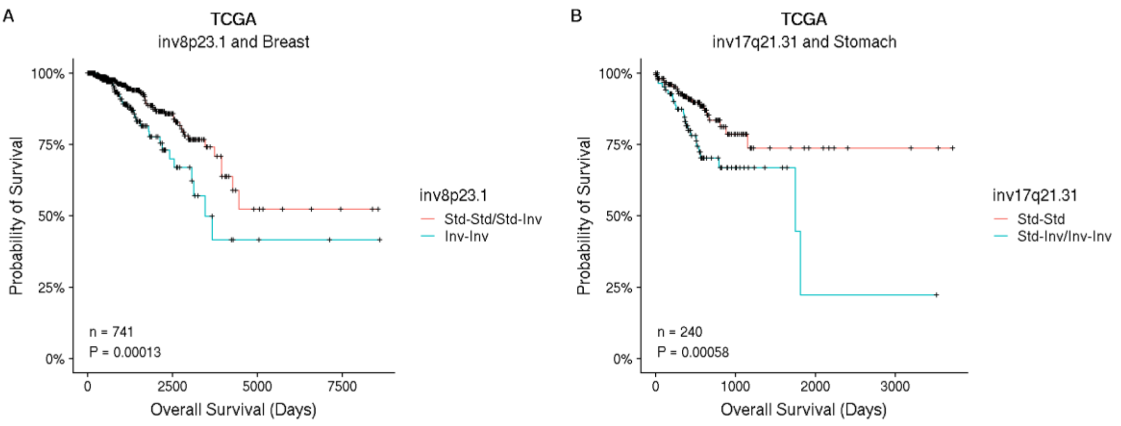

Supplementary Figure 1: Effect of inversions on cancer prognosis. A: Overall survival in TCGA Breast cancer for inversion inv8p23.1 using the recessive model (Std-Std and Std-Inv vs Inv-Inv). B: Overall survival in TCGA Stomach cancer for inversion inv17q21.31 using the dominant model (Std-Std vs Std-Inv and Inv-Inv).

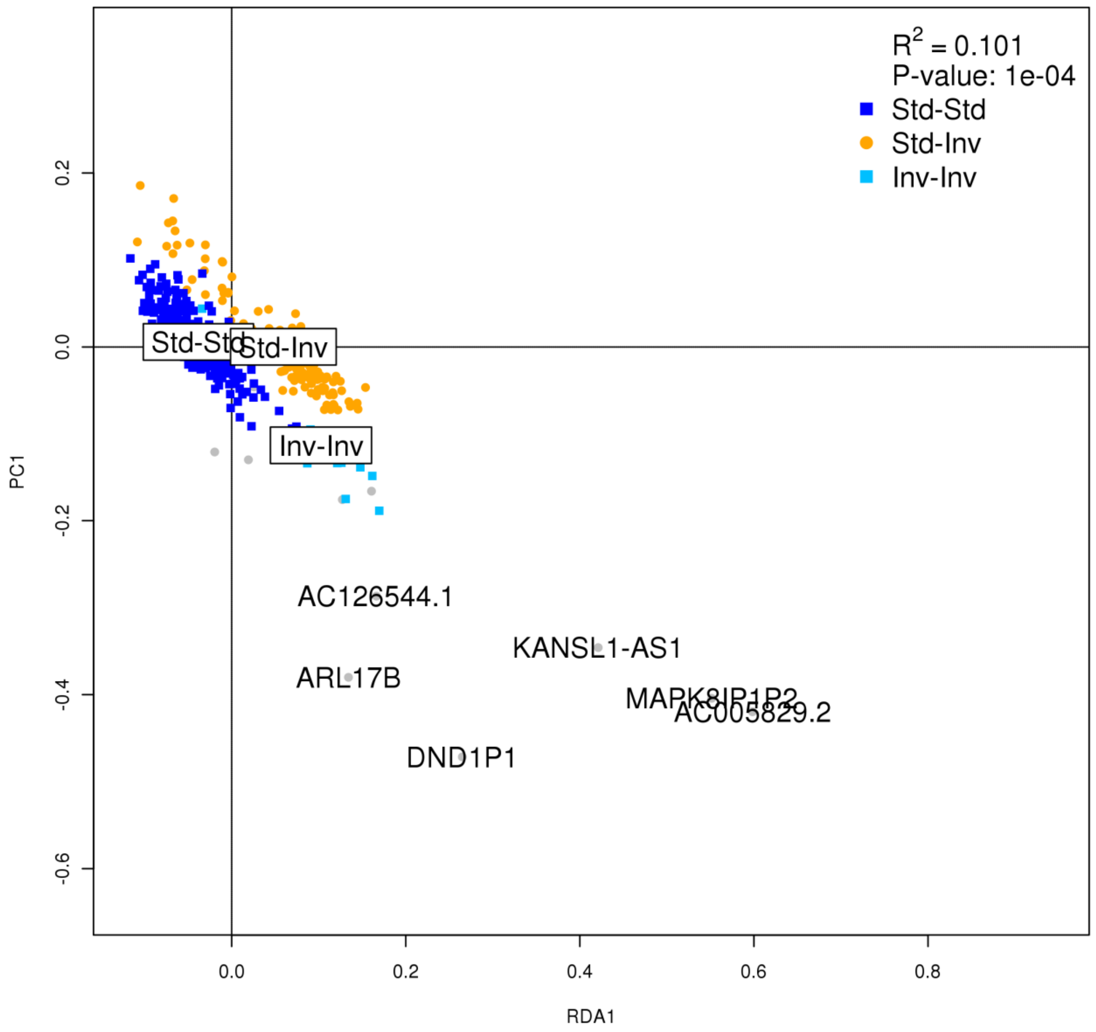

Supplementary Figure 2: Redundancy analysis of inversion inv17q21.31 on gene expression in colorectal cancer. Labels are the genes more associated to first RDA and first PC component. The results indicates that the inversion explain 10.1% of the total variability observed in gene expression of transcripts located in the 17q21.31 region (p<0.0001). Boxes represent the centroid of individuals belonging to each inversion genotype.

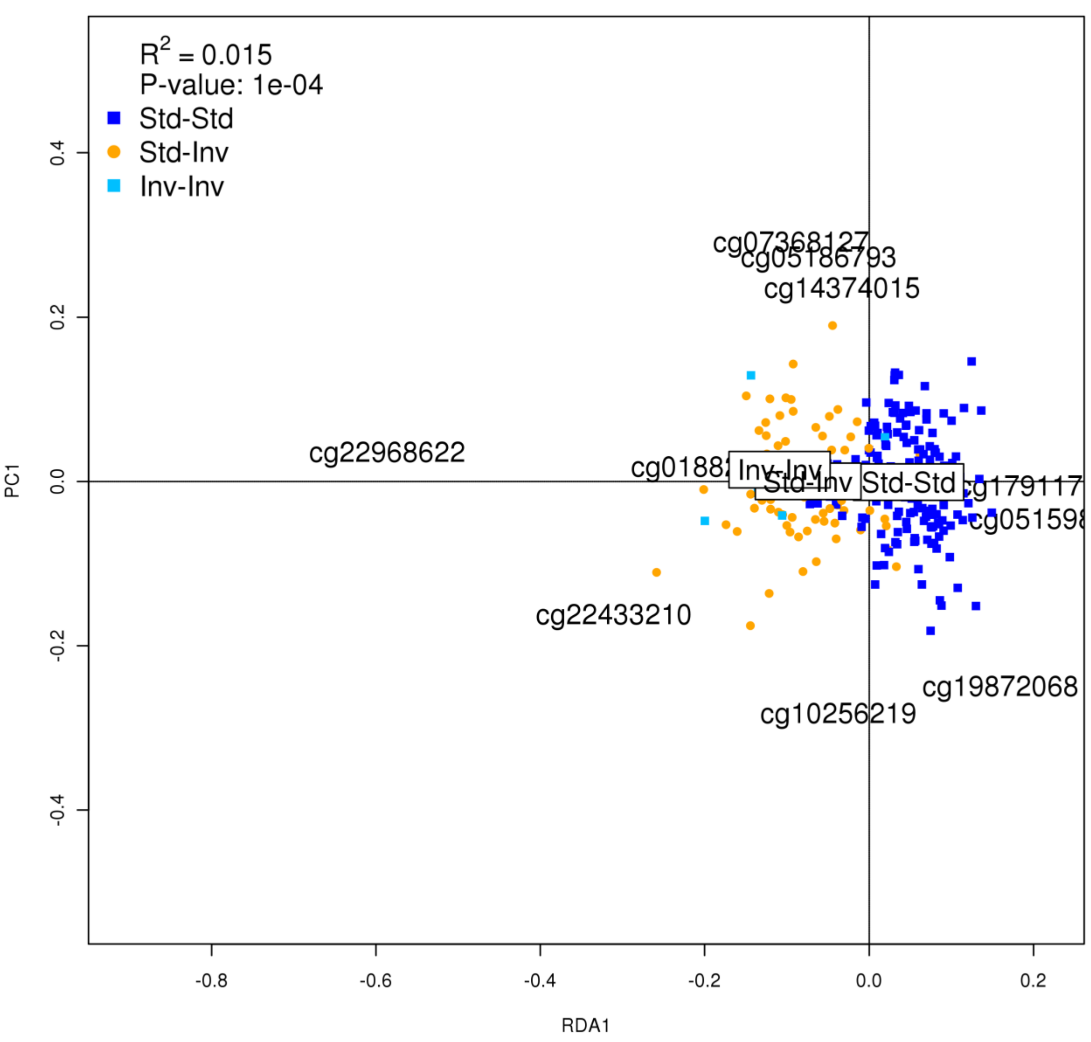

Supplementary Figure 3: Redundancy analysis of inversion inv17q21.31 on DNA methylation in colorectal cancer. Labels are the CpGs more associated to first RDA and first PC component. The results indicates that the inversion explain 1.5% of the total variability observed in DNA methylation located in the 17q21.31 region (p<0.0001). Boxes represent the centroid of individuals belonging to each inversion genotype.

Supplementary Table 1: Adjusted Crude Cox regression models between chromosomal inversions and overall survival in breast cancer. Breast cancer corresponds to BRCA dataset in TCGA. In bold, variables significantly associated to overall survival.

	inv8p23.1 (Recessive)		inv17q21.31 (Additive)	
	HR (95% CI)	p-value	HR (95% CI)	p-value
Inversion	2.55 (1.58-4.13)	$1.37 \cdot 10^{-4}$	1.36 (0.93-1.99)	0.11
Age	1.04 (1.02-1.06)	$8.81 \cdot 10^{-5}$	1.04 (1.02-1.06)	$1.92 \cdot 10^{-4}$
Stage II	1.61 (0.78-3.32)	0.20	1.54 (0.75-3.17)	0.24
Stage III	3.29 (1.51-7.17)	$2.66 \cdot 10^{-3}$	3.13 (1.44-6.79)	$3.96 \cdot 10^{-3}$
Stage IV	10.84 (4.46-26.33)	$1.41 \cdot 10^{-7}$	8.74 (3.62-21.09)	$1.40 \cdot 10^{-6}$
PC1	0.48 (0.07-3.32)	0.45	0.40 (0.06-2.89)	0.37
PC2	0.86 (0.15-4.94)	0.86	0.91 (0.16-5.14)	0.91
PC3	0.34 (0.11-1.05)	0.06	0.38 (0.12-1.19)	0.10
PC4	0.37 (0.11-1.21)	0.10	0.55 (0.16-1.83)	0.33

Supplementary Table 2: Adjusted Crude Cox regression models between chromosomal inversions and overall survival in stomach cancer. Stomach cancer corresponds to STAD dataset in TCGA. In bold, variables significantly associated to overall survival.

	inv8p23.1 (Overdominant)		inv17q21.31 (Dominant)	
	HR (95% CI)	p-value	HR (95% CI)	p-value
Inversion	1.61 (0.85-3.06)	0.14	**3.26 (1.66-6.39)**	$\mathbf{5.80 \cdot 10^{-4}}$
Age	**1.04 (1.00-1.07)**	**0.04**	**1.04 (1.01-1.08)**	$\mathbf{9.41 \cdot 10^{-3}}$
Sex	0.79 (0.41-1.51)	0.47	0.59 (0.30-1.15)	0.12
Stage II	**0.31 (0.10-0.96)**	**0.04**	**0.29 (0.09-0.90)**	**0.03**
Stage III	0.81 (0.34-1.92)	0.63	0.83 (0.35-1.98)	0.67
Stage IV	**3.04 (1.08-8.53)**	**0.03**	**3.10 (1.10-8.79)**	**0.03**
PC1	2.56 (0.11-58.55)	0.56	1.47 (0.07-31.75)	0.81
PC2	0.15 (0.01-1.86)	0.14	0.07 (0.01-0.95)	0.05
PC3	0.74 (0.15-3.61)	0.71	0.57 (0.12-2.63)	0.47
PC4	0.80 (0.13-4.82)	0.81	0.97 (0.16-5.76)	0.97

Supplementary Table 3: Adjusted Crude Cox regression models between chromosomal inversions and overall survival in lung adenocarcinoma. Lung adenocarcinoma corresponds to LUAD dataset in TCGA. In bold, variables significantly associated to overall survival.

	inv8p23.1 (Overdominant)		inv17q21.31 (Dominant)	
	HR (95% CI)	p-value	HR (95% CI)	p-value
Inversion	1.08 (0.70-1.65)	0.73	0.95 (0.61-1.48)	0.82
Age	**1.03 (1.00-1.05)**	**0.04**	1.02 (1.00-1.05)	0.05
Sex	0.89 (0.58-1.37)	0.60	0.89 (0.57-1.38)	0.60
Stage II	**2.20 (1.27-3.81)**	**$4.67 \cdot 10^{-3}$**	**2.18 (1.26-3.78)**	**$5.49 \cdot 10^{-3}$**
Stage III	**4.38 (2.63-7.28)**	**$1.23 \cdot 10^{-8}$**	**4.37 (2.63-7.27)**	**$1.32 \cdot 10^{-8}$**
Stage IV	**4.18 (1.86-9.41)**	**$5.54 \cdot 10^{-4}$**	**4.05 (1.81-9.03)**	**$6.41 \cdot 10^{-4}$**
PC1	0.31 (0.05-1.83)	0.20	0.31 (0.05-1.84)	0.19
PC2	0.81 (0.17-3.91)	0.79	0.82 (0.17-3.96)	0.80
PC3	0.65 (0.22-1.89)	0.43	0.64 (0.22-1.87)	0.42
PC4	0.68 (0.25-1.82)	0.44	0.67 (0.25-1.80)	0.43

Supplementary Table 4: Adjusted Crude Cox regression models between chromosomal inversions and overall survival in lung squamous cell carcinoma. Lung squamous cell carcinoma corresponds to LUSC dataset in TCGA. In bold, variables significantly associated to overall survival.

	inv8p23.1 (Overdominant)		inv17q21.31 (Recessive)	
	HR (95% CI)	p-value	HR (95% CI)	p-value
Inversion	1.11 (0.78-1.6)	0.56	0.64 (0.29-1.38)	0.25
Age	1.02 (1.00-1.05)	0.08	1.02 (1.00-1.04)	0.09
Sex	1.16 (0.76-1.76)	0.49	1.16 (0.76-1.77)	0.48
Stage II	1.21 (0.80-1.84)	0.37	1.21 (0.80-1.84)	0.37
Stage III	**1.66 (1.05-2.64)**	**0.03**	**1.64 (1.04-2.59)**	**0.03**
Stage IV	2.72 (0.65-11.33)	0.17	2.60 (0.63-10.82)	0.19
PC1	**8.63 (1.81-41.1)**	**$6.81 \cdot 10^{-3}$**	**8.50 (1.77-40.76)**	**$7.48 \cdot 10^{-3}$**
PC2	0.81 (0.19-3.44)	0.77	0.73 (0.17-3.17)	0.68
PC3	0.46 (0.18-1.2)	0.11	0.45 (0.18-1.16)	0.10
PC4	1.15 (0.44-3.04)	0.78	1.10 (0.41-2.90)	0.85

Supplementary Table 5: Adjusted Crude Cox regression models between chromosomal inversions and overall survival in liver cancer. Liver cancer corresponds to LIHC dataset in TCGA. In bold, variables significantly associated to overall survival.

	inv8p23.1 (Dominant)		inv17q21.31 (Overdominant)	
	HR (95% CI)	p-value	HR (95% CI)	p-value
Inversion	0.84 (0.38-1.87)	0.67	0.79 (0.41-1.51)	0.47
Age	1.02 (0.99-1.05)	0.13	1.02 (0.99-1.05)	0.11
Sex	0.95 (0.51-1.78)	0.87	1.01 (0.53-1.92)	0.97
Stage II	0.79 (0.35-1.80)	0.57	0.84 (0.38-1.85)	0.67
Stage III	1.42 (0.72-2.78)	0.31	1.42 (0.73-2.77)	0.31
Stage IV	2.78 (0.59-13.06)	0.20	2.73 (0.59-12.70)	0.20
PC1	2.43 (0.21-28.10)	0.48	2.09 (0.19-23.61)	0.55
PC2	**0.04 (0.00-0.53)**	**0.02**	**0.03 (0.00-0.46)**	**0.01**
PC3	3.74 (0.79-17.71)	0.10	3.89 (0.82-18.37)	0.09
PC4	2.90 (0.67-12.60)	0.16	2.44 (0.58-10.30)	0.22

Supplementary Table 6: Adjusted Crude Cox regression models between chromosomal inversions and overall survival in colorectal cancer. Colorectal corresponds to the merge of COAD and READ datasets in TCGA. In bold, variables significantly associated to overall survival.

	inv8p23.1 (Recessive)		inv17q21.31 (Overdominant)	
	HR (95% CI)	p-value	HR (95% CI)	p-value
Inversion	1.77 (0.96-3.26)	0.07	0.75 (0.39-1.45)	0.40
Age	**1.05 (1.02-1.08)**	**$6.71 \cdot 10^{-4}$**	**1.05 (1.02-1.08)**	**$5.95 \cdot 10^{-4}$**
Sex	1.12 (0.62-2.03)	0.71	1.15 (0.64-2.07)	0.65
Stage II	1.31 (0.43-3.96)	0.63	1.29 (0.42-3.95)	0.65
Stage III	1.89 (0.60-5.98)	0.28	1.62 (0.50-5.22)	0.42
Stage IV	**6.34 (2.05-19.59)**	**$1.32 \cdot 10^{-3}$**	**5.37 (1.73-16.67)**	**$3.61 \cdot 10^{-3}$**
PC1	6.27 (0.55-70.96)	0.14	5.82 (0.54-62.88)	0.15
PC2	0.31 (0.03-3.63)	0.35	0.30 (0.03-3.53)	0.34
PC3	3.42 (0.98-12.00)	0.05	3.41 (0.96-12.07)	0.06
PC4	0.38 (0.08-1.78)	0.22	0.41 (0.09-1.93)	0.26

Supplementary Table 7: Adjusted Crude Cox regression models between chromosomal inversions and disease-free survival in colorectal cancer. Colorectal corresponds to the merge of COAD and READ datasets in TCGA. In bold, variables significantly associated to disease-free survival.

	inv8p23.1 (Overdominant)		inv17q21.31 (Overdominant)	
	HR (95% CI)	p-value	HR (95% CI)	p-value
Inversion	0.80 (0.52-1.24)	0.32	**1.81 (1.17-2.78)**	**$7.21 \cdot 10^{-3}$**
Age	0.99 (0.98-1.01)	0.60	0.99 (0.98-1.01)	0.56
Sex	1.19 (0.77-1.85)	0.43	1.25 (0.81-1.94)	0.31
Stage II	1.59 (0.69-3.66)	0.28	1.86 (0.80-4.30)	0.15
Stage III	**2.51 (1.09-5.81)**	**0.03**	**2.94 (1.26-6.85)**	**0.01**
Stage IV	**9.63 (4.15-22.35)**	**$1.36 \cdot 10^{-7}$**	**10.61 (4.56-24.70)**	**$4.34 \cdot 10^{-8}$**
PC1	1.92 (0.31-11.96)	0.49	1.95 (0.30-12.59)	0.48
PC2	1.56 (0.28-8.80)	0.62	1.74 (0.31-9.87)	0.53
PC3	1.34 (0.51-3.54)	0.55	1.44 (0.55-3.77)	0.46
PC4	0.64 (0.22-1.92)	0.43	0.64 (0.22-1.88)	0.41

Supplementary Table 8: Adjusted Crude Cox regression models between chromosomal inversions and disease-free survival in breast cancer. Breast cancer corresponds to BRCA dataset in TCGA. In bold, variables significantly associated to disease-free survival.

	inv8p23.1 (Overdominant)		inv17q21.31 (Recessive)	
	HR (95% CI)	p-value	HR (95% CI)	p-value
Inversion	**0.58 (0.36-0.93)**	**0.02**	1.90 (0.73-4.93)	0.19
Age	1.01 (0.99-1.03)	0.56	1.01 (0.99-1.03)	0.45
Stage II	1.38 (0.65-2.93)	0.40	1.37 (0.65-2.91)	0.41
Stage III	**3.52 (1.63-7.61)**	**$1.34 \cdot 10^{-3}$**	**3.27 (1.52-7.07)**	**$2.52 \cdot 10^{-3}$**
Stage IV	**8.66 (3.13-23.97)**	**$3.25 \cdot 10^{-5}$**	**7.98 (2.90-22.00)**	**$5.92 \cdot 10^{-5}$**
PC1	2.85 (0.37-22.14)	0.32	3.63 (0.46-28.83)	0.22
PC2	1.35 (0.23-7.83)	0.74	1.20 (0.21-6.89)	0.84
PC3	0.60 (0.18-2.00)	0.40	0.70 (0.21-2.35)	0.57
PC4	0.52 (0.16-1.64)	0.26	0.53 (0.17-1.66)	0.28

Supplementary Table 9: Adjusted Crude Cox regression models between chromosomal inversions and disease-free survival in stomach cancer. Stomach cancer corresponds to STAD dataset in TCGA. In bold, variables significantly associated to disease-free survival.

	inv8p23.1 (Overdominant)		inv17q21.31 (Overdominant)	
	HR (95% CI)	p-value	HR (95% CI)	p-value
Inversion	1.24 (0.73-2.09)	0.43	1.07 (0.61-1.88)	0.81
Age	0.99 (0.97-1.02)	0.62	0.99 (0.97-1.02)	0.63
Sex	1.50 (0.82-2.72)	0.19	1.47 (0.80-2.7)	0.21
Stage II	1.23 (0.45-3.30)	0.69	1.22 (0.45-3.31)	0.69
Stage III	**2.53 (1.04-6.15)**	**0.04**	**2.55 (1.05-6.18)**	**0.04**
Stage IV	**4.20 (1.38-12.77)**	**0.01**	**4.12 (1.36-12.51)**	**0.01**
PC1	0.26 (0.02-2.80)	0.26	0.29 (0.03-3.13)	0.31
PC2	0.16 (0.02-1.22)	0.08	0.16 (0.02-1.20)	0.07
PC3	0.68 (0.17-2.74)	0.59	0.66 (0.17-2.62)	0.55
PC4	0.48 (0.12-2.03)	0.32	0.47 (0.11-1.99)	0.31

Supplementary Table 10: Adjusted Crude Cox regression models between chromosomal inversions and disease-free survival in lung adenocarcinoma. Lung adenocarcinoma corresponds to LUAD dataset in TCGA. In bold, variables significantly associated to disease-free survival.

	inv8p23.1 (Additive)		inv17q21.31 (Dominant)	
	HR (95% CI)	p-value	HR (95% CI)	p-value
Inversion	**0.71 (0.54-0.94)**	**0.01**	0.94 (0.66-1.32)	0.71
Age	1.01 (0.99-1.03)	0.29	1.01 (0.99-1.03)	0.33
Sex	1.18 (0.83-1.68)	0.36	1.20 (0.84-1.70)	0.32
Stage II	**2.27 (1.53-3.38)**	**$4.94 \cdot 10^{-5}$**	**2.08 (1.40-3.09)**	**$2.63 \cdot 10^{-4}$**
Stage III	**2.26 (1.41-3.60)**	**$6.48 \cdot 10^{-4}$**	**2.15 (1.35-3.42)**	**$1.30 \cdot 10^{-3}$**
Stage IV	0.89 (0.28-2.89)	0.85	0.96 (0.30-3.11)	0.95
PC1	0.39 (0.09-1.65)	0.20	0.34 (0.08-1.45)	0.15
PC2	1.40 (0.40-4.92)	0.60	1.35 (0.39-4.70)	0.64
PC3	1.62 (0.65-4.00)	0.30	1.73 (0.70-4.27)	0.23
PC4	0.84 (0.38-1.88)	0.68	0.80 (0.36-1.78)	0.59

Supplementary Table 11: Adjusted Crude Cox regression models between chromosomal inversions and disease-free survival in lung squamous cell carcinoma. Lung squamous cell carcinoma corresponds to LUSC dataset in TCGA. In bold, variables significantly associated to disease-free survival.

	inv8p23.1 (Additive)		inv17q21.31 (Recessive)	
	HR (95% CI)	p-value	HR (95% CI)	p-value
Inversion	0.92 (0.70-1.22)	0.56	0.39 (0.14-1.09)	0.07
Age	1.01 (0.98-1.03)	0.49	1.01 (0.99-1.03)	0.47
Sex	1.13 (0.72-1.79)	0.59	1.13 (0.72-1.78)	0.59
Stage II	**1.68 (1.07-2.64)**	**0.02**	**1.76 (1.12-2.77)**	**0.01**
Stage III	**2.25 (1.35-3.75)**	**$1.87\cdot10^{-3}$**	**2.27 (1.36-3.78)**	**$1.65\cdot10^{-3}$**
Stage IV	1.70 (0.23-12.48)	0.60	1.58 (0.22-11.58)	0.65
PC1	1.84 (0.32-10.48)	0.49	1.77 (0.31-10.06)	0.52
PC2	1.99 (0.40-9.85)	0.40	1.92 (0.38-9.62)	0.43
PC3	0.60 (0.22-1.64)	0.32	0.58 (0.21-1.59)	0.29
PC4	1.10 (0.38-3.17)	0.86	1.01 (0.35-2.90)	0.99

Supplementary Table 12: Adjusted Crude Cox regression models between chromosomal inversions and disease-free survival in liver cancer. Liver cancer corresponds to LIHC dataset in TCGA. In bold, variables significantly associated to disease-free survival.

	inv8p23.1 (Overdominant)		inv17q21.31 (Dominant)	
	HR (95% CI)	p-value	HR (95% CI)	p-value
Inversion	1.20 (0.70-2.06)	0.50	1.13 (0.70-1.83)	0.62
Age	0.99 (0.97-1.01)	0.41	0.99 (0.97-1.01)	0.28
Sex	0.69 (0.40-1.18)	0.17	0.67 (0.39-1.14)	0.14
Stage II	**2.35 (1.27-4.36)**	**$6.49\cdot10^{-3}$**	**2.24 (1.22-4.13)**	**$9.75\cdot10^{-3}$**
Stage III	**3.34 (1.78-6.26)**	**$1.64\cdot10^{-4}$**	**3.13 (1.72-5.69)**	**$1.88\cdot10^{-4}$**
Stage IV	4.14 (0.90-18.98)	0.07	4.10 (0.89-18.91)	0.07
PC1	5.86 (0.71-48.01)	0.10	5.83 (0.69-49.25)	0.11
PC2	**0.01 (0.00-0.13)**	**$2.09\cdot10^{-4}$**	**0.01 (0.00-0.13)**	**$1.99\cdot10^{-4}$**
PC3	2.01 (0.60-6.72)	0.26	1.98 (0.59-6.70)	0.27
PC4	0.62 (0.21-1.86)	0.39	0.69 (0.24-2.01)	0.50

Supplementary Table 13: Participant features in CRCGEN. Continuous variables are described with median and range. Categorical variables are described with counts and percentage of each category. In bold, participant features different to TCGA colorectal dataset.

	CRCGEN (n = 760)
**inv17q21.31**	
Std-Std	402 (52.9%)
Std-Inv	311 (40.9%)
InvInv	47 (6.2%)
Age (years)	68 (23-91)
**Sex**	
Women	276 (36.3%)
Men	484 (63.7%)
**Pathologic stage**	
Stage I	87 (11.4%)
Stage II	248 (32.6%)
Stage III	316 (41.6%)
Stage IV	109 (14.4%)
City	
Barcelona	596 (78.4%)
León	164 (21.6%)
Study	
Barcelona 1	287 (40.7%)
Barcelona 2	309 (37.7%)
León	164 (21.6%)

Supplementary Table 14: Cox regression models between inv17q21.31 and colorectal disease-free survival in CRCGEN.

	Univariate	
	HR (95% CI)	p-value
inv17q21.31	1.16 (0.85-1.59)	0.33
	**Multivariate**	
	HR (95% CI)	p-value
inv17q21.31	1.14 (0.84-1.56)	0.40
Age	1.00 (0.99-1.01)	0.99
Sex	1.00 (0.73-1.39)	0.96
Stage II	1.85 (0.78-4.39)	0.16
Stage III	**3.75 (1.63-8.62)**	**$1.8 \cdot 10^{-3}$**
Stage IV	**5.51 (2.26-8.62)**	**$1.8 \cdot 10^{-4}$**
City	1.04 (0.71-1.52)	0.84

Supplementary Table 15: Genes modified by inversion inv17q21.31. Genes in bold are located in the inversion region. log2FC: differences in gene expression between inversion heterozygous and inversion homozygous (i.e overdominant model). Average Expression: Average expression of the gene in log2CPM Adj p-value: p-value adjusted by B&H. Coordinates: gene coordinates in hg19.

Gene Symbol	log_2FC	Average Expression	p-value	Adj p-value	Coordinates
**AC005829.2**	**4.62**	**-3.11**	**$1.35 \cdot 10^{-136}$**	**$3.76 \cdot 10^{-132}$**	**chr17:44336917-44337972**
**MAPK8IP1P2**	**3.41**	**-4.30**	**$5.68 \cdot 10^{-128}$**	**$7.93 \cdot 10^{-124}$**	**chr17:43678235-43679706**
**KANSL1-AS1**	**1.44**	**0.85**	**$2.67 \cdot 10^{-73}$**	**$2.49 \cdot 10^{-69}$**	**chr17:44270942-44274089**
**LRRC37A4P**	**-1.01**	**1.20**	**$1.50 \cdot 10^{-60}$**	**$1.05 \cdot 10^{-56}$**	**chr17:43578685-43627701**
**DND1P1**	**0.91**	**-0.34**	**$1.82 \cdot 10^{-33}$**	**$1.02 \cdot 10^{-29}$**	**chr17:43663237-43664295**
**LRRC37A2**	**0.40**	**1.26**	**$4.97 \cdot 10^{-17}$**	**$2.31 \cdot 10^{-13}$**	**chr17:44588877-44633016**
**LINC02210**	**0.24**	**3.61**	**$5.03 \cdot 10^{-16}$**	**$2.01 \cdot 10^{-12}$**	**chr17:43697694-43725582**
**AC126544.1**	**0.68**	**-2.66**	**$1.41 \cdot 10^{-12}$**	**$4.91 \cdot 10^{-9}$**	**chr17:43669987-43670735**
**ARL17A**	**0.36**	**-1.04**	**$1.26 \cdot 10^{-8}$**	**$3.90 \cdot 10^{-5}$**	**chr17:44594068-44657088**
**AC005829.1**	**0.41**	**-2.15**	**$3.32 \cdot 10^{-7}$**	**$9.26 \cdot 10^{-4}$**	**chr17:44344403-44346060**
**AC091132.5**	**-0.52**	**-3.40**	**$9.55 \cdot 10^{-7}$**	**$2.43 \cdot 10^{-3}$**	**chr17:43623170-43640596**
TM4SF19	-0.47	-2.45	$4.33 \cdot 10^{-6}$	$1.01 \cdot 10^{-2}$	chr3:196046213-196065374
PTP4A2	-0.09	7.69	$6.69 \cdot 10^{-6}$	$1.44 \cdot 10^{-2}$	chr1:32372022-32410457
GCM1	0.61	-3.72	$1.27 \cdot 10^{-5}$	$2.53 \cdot 10^{-2}$	chr6:52991762-53013627
ERLIN2	-0.17	6.26	$1.52 \cdot 10^{-5}$	$2.82 \cdot 10^{-2}$	chr8:37594117-37616619
PLBD2	-0.14	6.20	$2.30 \cdot 10^{-5}$	$3.81 \cdot 10^{-2}$	chr12:113796371-113827203
**ARL17B**	**0.40**	**-1.49**	**$2.32 \cdot 10^{-5}$**	**$3.81 \cdot 10^{-2}$**	**chr17:44352150-44439130**

Supplementary Table 16: CpGs affected by inversion inv17q21.31 in colorectal cancer. In bold, CpGs in the inversion region. beta: differences in DNA methylation between inversion heterozygous and inversion homozygous. Ave Meth: Average methylation of the CpGs in beta values. Adj p-value: p-value adjusted by B&H. Coordinates: gene coordinates in hg19. Gene Symbol: Name of the gene where the CpG is located. Gene Position: position inside the gene where the CpG is located.

CpG	Beta	Ave Meth	p-value	Adj p-value	Coordinates	Gene Symbol	Gene Position
**cg22968622**	**0.435**	**0.175**	**$1.10\cdot10^{-251}$**	**$3.87\cdot10^{-246}$**	**chr17:43663579**		
**cg22433210**	**0.003**	**0.02**	**$8.17\cdot10^{-21}$**	**$1.43\cdot10^{-15}$**	**chr17:43662623**		
**cg01341218**	**0.004**	**0.023**	**$6.61\cdot10^{-16}$**	**$7.74\cdot10^{-11}$**	**chr17:43662625**		
**cg05159804**	**-0.084**	**0.296**	**$4.93\cdot10^{-14}$**	**$4.32\cdot10^{-09}$**	**chr17:44343776**		
**cg17911788**	**-0.164**	**0.356**	**$3.51\cdot10^{-13}$**	**$2.47\cdot10^{-08}$**	**chr17:44343683**		
**cg01882395**	**0.018**	**0.085**	**$1.65\cdot10^{-10}$**	**$9.63\cdot10^{-06}$**	**chr17:43717810**	**LINC02210**	**Body**
cg15264255	0.005	0.045	$1.92\cdot10^{-09}$	$9.62\cdot10^{-05}$	chr8:99306718	NIPAL2	TSS200
cg03084996	0.043	0.296	$4.92\cdot10^{-08}$	$2.16\cdot10^{-03}$	chr8:69539427	C8orf34	Body
**cg24477401**	**-0.022**	**0.262**	**$1.82\cdot10^{-07}$**	**$6.51\cdot10^{-03}$**	**chr17:43484506**	**ARHGAP27**	**TSS1500/5'UTR**
cg18012760	-0.092	0.476	$1.85\cdot10^{-07}$	$6.51\cdot10^{-03}$	chr5:6449577	UBE2QL1	Body
cg11970204	0.03	0.813	$2.37\cdot10^{-07}$	$7.55\cdot10^{-03}$	chr10:103245907	BTRC	Body
cg03450509	0.004	0.033	$3.53\cdot10^{-07}$	$1.03\cdot10^{-02}$	chr8:37654283	ADGRA2	TSS200
cg08684639	0.089	0.487	$6.80\cdot10^{-07}$	$1.83\cdot10^{-02}$	chr4:176987313	WDR17	5'UTR
cg02401614	0.014	0.897	$8.74\cdot10^{-07}$	$1.89\cdot10^{-02}$	chr3:182509366		
**cg04491389**	**-0.011**	**0.924**	**$9.02\cdot10^{-07}$**	**$1.89\cdot10^{-02}$**	**chr17:44214771**	**KANSL1**	**Body**
cg06610368	0.013	0.069	$9.05\cdot10^{-07}$	$1.89\cdot10^{-02}$	chr5:86564371	RASA1	1stExon/TSS1500
cg26896668	0.057	0.188	$9.71\cdot10^{-07}$	$1.89\cdot10^{-02}$	chr10:28957429		
cg09416109	0.013	0.874	$9.46\cdot10^{-07}$	$1.89\cdot10^{-02}$	chr16:57416332	CX3CL1	Body
cg11807006	-0.024	0.167	$1.10\cdot10^{-06}$	$2.03\cdot10^{-02}$	chr17:7239821	ACAP1	TSS200
cg21073637	0.004	0.046	$1.24\cdot10^{-06}$	$2.18\cdot10^{-02}$	chr14:64010844	PPP2R5E	TSS1500
cg26943759	-0.035	0.814	$1.61\cdot10^{-06}$	$2.49\cdot10^{-02}$	chr16:87100606		
cg22367989	0.01	0.063	$1.86\cdot10^{-06}$	$2.49\cdot10^{-02}$	chr2:206546660	NRP2	TSS1500
cg06733329	0.01	0.088	$1.71\cdot10^{-06}$	$2.49\cdot10^{-02}$	chr5:176740039	MXD3	TSS1500
cg21140943	0.013	0.086	$1.87\cdot10^{-06}$	$2.49\cdot10^{-02}$	chr15:93631110	RGMA	Body/5'UTR
cg05797660	0.006	0.061	$1.82\cdot10^{-06}$	$2.49\cdot10^{-02}$	chr8:96145761	PLEKHF2	TSS1500
cg07240557	-0.028	0.251	$1.87\cdot10^{-06}$	$2.49\cdot10^{-02}$	chr12:27396937	STK38L	TSS200
cg07685869	0.015	0.097	$1.97\cdot10^{-06}$	$2.49\cdot10^{-02}$	chr16:57836706	KIFC3	TSS1500
cg27021131	0.006	0.045	$2.02\cdot10^{-06}$	$2.49\cdot10^{-02}$	chr16:53737924	RPGRIP1L<br>FTO	TSS200<br>1stExon/5'UTR
cg22567473	0.005	0.037	$2.05\cdot10^{-06}$	$2.49\cdot10^{-02}$	chr15:63902661	HERC1	Body
cg00876127	0.046	0.309	$2.15\cdot10^{-06}$	$2.50\cdot10^{-02}$	chr16:56554313	BBS2	TSS1500
cg02473439	-0.052	0.819	$2.21\cdot10^{-06}$	$2.50\cdot10^{-02}$	chr10:70515244	CCAR1	Body
cg25150846	-0.004	0.964	$2.47\cdot10^{-06}$	$2.67\cdot10^{-02}$	chr11:2189591	TH	Body
cg23624957	-0.044	0.463	$2.51\cdot10^{-06}$	$2.67\cdot10^{-02}$	chr6:114257531	HDAC2	3'UTR
cg14659771	0.005	0.038	$2.88\cdot10^{-06}$	$2.90\cdot10^{-02}$	chr2:231917362		
cg16592223	-0.056	0.662	$2.89\cdot10^{-06}$	$2.90\cdot10^{-02}$	chr16:29144962		
cg20615848	0.054	0.339	$3.14\cdot10^{-06}$	$3.06\cdot10^{-02}$	chr18:31020973	CCDC178	TSS1500
cg18823272	-0.021	0.895	$3.48\cdot10^{-06}$	$3.23\cdot10^{-02}$	chr19:34111733	CHST8	TSS1500

cg06009422	-0.061	0.565	$3.50 \cdot 10^{-06}$	$3.23 \cdot 10^{-02}$	chr6:161656403	AGPAT4	5'UTR
cg05807467	0.031	0.705	$3.81 \cdot 10^{-06}$	$3.36 \cdot 10^{-02}$	chr8:17579494	MTUS1	1stExon/5'UTR/Body
cg20132549	0.004	0.046	$3.83 \cdot 10^{-06}$	$3.36 \cdot 10^{-02}$	chr11:442189	ANO9	TSS200
cg27537125	0.048	0.267	$4.05 \cdot 10^{-06}$	$3.47 \cdot 10^{-02}$	chr1:25349681		
cg03514660	-0.048	0.339	$4.17 \cdot 10^{-06}$	$3.48 \cdot 10^{-02}$	chr12:131418057		
cg25265234	-0.036	0.817	$4.39 \cdot 10^{-06}$	$3.58 \cdot 10^{-02}$	chr3:106955668	LINC00882	Body
cg12528597	0.068	0.719	$4.93 \cdot 10^{-06}$	$3.85 \cdot 10^{-02}$	chr11:57232556	RTN4RL2	Body
cg18219994	0.012	0.859	$5.08 \cdot 10^{-06}$	$3.85 \cdot 10^{-02}$	chr8:140826557	TRAPPC9	Body
cg13471990	-0.037	0.229	$5.11 \cdot 10^{-06}$	$3.85 \cdot 10^{-02}$	chr10:97515222	ENTPD1	TSS1500/TSS200/Body
cg20136855	0.025	0.693	$5.26 \cdot 10^{-06}$	$3.85 \cdot 10^{-02}$	chr1:12233852	TNFRSF1B	Body
cg11941929	0.017	0.778	$5.19 \cdot 10^{-06}$	$3.85 \cdot 10^{-02}$	chr18:12429120	PRELID3A	Body
**cg07368061**	**-0.046**	**0.754**	$\mathbf{5.63 \cdot 10^{-06}}$	$\mathbf{4.02 \cdot 10^{-02}}$	**chr17:44090862**	**MAPT**	**Body**
cg07707875	0.016	0.081	$5.87 \cdot 10^{-06}$	$4.02 \cdot 10^{-02}$	chr7:155326102	CNPY1	5'UTR
cg08283464	-0.041	0.557	$6.15 \cdot 10^{-06}$	$4.02 \cdot 10^{-02}$	chr10:132877148		
cg13003311	-0.029	0.206	$6.45 \cdot 10^{-06}$	$4.02 \cdot 10^{-02}$	chr5:89853284	ADGRV1	TSS1500
cg11620409	0.029	0.164	$6.35 \cdot 10^{-06}$	$4.02 \cdot 10^{-02}$	chr11:61519687	MYRF	TSS1500
cg12743248	0.022	0.198	$6.37 \cdot 10^{-06}$	$4.02 \cdot 10^{-02}$	chr8:49636840	EFCAB1	3'UTR/Body
cg12075720	-0.084	0.475	$6.55 \cdot 10^{-06}$	$4.02 \cdot 10^{-02}$	chr19:17799037	UNC13A	TSS200
cg26127816	0.016	0.797	$6.55 \cdot 10^{-06}$	$4.02 \cdot 10^{-02}$	chr8:144872260		
cg25010832	-0.057	0.634	$6.67 \cdot 10^{-06}$	$4.02 \cdot 10^{-02}$	chr5:178634615	ADAMTS2	Body
cg05152300	-0.065	0.544	$6.73 \cdot 10^{-06}$	$4.02 \cdot 10^{-02}$	chr16:17458534	XYLT1	Body
cg17954226	0.073	0.397	$6.76 \cdot 10^{-06}$	$4.02 \cdot 10^{-02}$	chr10:26846020	APBB1IP	Body
cg03999934	-0.077	0.449	$7.06 \cdot 10^{-06}$	$4.06 \cdot 10^{-02}$	chr6:30325790		
cg09938674	-0.074	0.582	$7.00 \cdot 10^{-06}$	$4.06 \cdot 10^{-02}$	chr8:1534838	DLGAP2	Body
cg23280155	0.008	0.064	$8.23 \cdot 10^{-06}$	$4.45 \cdot 10^{-02}$	chr14:72219812		
**cg02301815**	**-0.009**	**0.929**	$\mathbf{7.86 \cdot 10^{-06}}$	$\mathbf{4.45 \cdot 10^{-02}}$	**chr17:44249491**	**KANSL1**	**1stExon**
cg19093405	-0.045	0.786	$8.33 \cdot 10^{-06}$	$4.45 \cdot 10^{-02}$	chr3:54807076	CACNA2D3	Body
cg27516159	0.046	0.363	$8.35 \cdot 10^{-06}$	$4.45 \cdot 10^{-02}$	chr12:1904847	CACNA2D4	Body
cg20410290	0.018	0.859	$8.38 \cdot 10^{-06}$	$4.45 \cdot 10^{-02}$	chr17:75786172		
cg06061238	-0.045	0.473	$8.60 \cdot 10^{-06}$	$4.50 \cdot 10^{-02}$	chr10:134915859	ADGRA1	Body
cg05359518	-0.058	0.691	$9.12 \cdot 10^{-06}$	$4.57 \cdot 10^{-02}$	chr15:57508437	TCF12	Body
cg15935121	-0.016	0.721	$8.99 \cdot 10^{-06}$	$4.57 \cdot 10^{-02}$	chr1:2230601	SKI	Body
cg22837763	0.056	0.324	$9.11 \cdot 10^{-06}$	$4.57 \cdot 10^{-02}$	chr8:108393034	ANGPT1	Body
cg27508281	-0.044	0.865	$9.59 \cdot 10^{-06}$	$4.74 \cdot 10^{-02}$	chr16:87027322		
cg03003256	-0.029	0.87	$9.97 \cdot 10^{-06}$	$4.81 \cdot 10^{-02}$	chr6:116849557	CALHM4 TRAPPC3L	TSS1500/Body
cg10345369	-0.028	0.852	$1.05 \cdot 10^{-05}$	$4.81 \cdot 10^{-02}$	chr10:131570053		
cg18460575	-0.039	0.814	$1.01 \cdot 10^{-05}$	$4.81 \cdot 10^{-02}$	chr10:131428401	MGMT	Body
cg09981407	-0.017	0.117	$1.02 \cdot 10^{-05}$	$4.81 \cdot 10^{-02}$	chr17:2699505	RAP1GAP2	TSS1500
cg17837330	0.032	0.254	$1.07 \cdot 10^{-05}$	$4.81 \cdot 10^{-02}$	chr8:130738823		
cg03538436	-0.048	0.619	$1.08 \cdot 10^{-05}$	$4.81 \cdot 10^{-02}$	chr12:117799370	NOS1	5'UTR/1stExon
cg24787130	0.05	0.389	$1.08 \cdot 10^{-05}$	$4.81 \cdot 10^{-02}$	chr5:146948683		
cg11040181	-0.074	0.484	$1.08 \cdot 10^{-05}$	$4.81 \cdot 10^{-02}$	chr8:2074935	MYOM2	Body
cg00288736	0.06	0.521	$1.11 \cdot 10^{-05}$	$4.84 \cdot 10^{-02}$	chr8:121140957	COL14A1	5'UTR
cg19177125	-0.023	0.369	$1.14 \cdot 10^{-05}$	$4.84 \cdot 10^{-02}$	chr18:19752414	GATA6	Body
cg27541604	0.056	0.371	$1.14 \cdot 10^{-05}$	$4.84 \cdot 10^{-02}$	chr1:159046451	AIM2	5'UTR/1stExon

cg12286194	0.002	0.024	$1.17 \cdot 10^{-05}$	$4.84 \cdot 10^{-02}$	chr8:126442431	TRIB1	TSS200
cg13451153	0.012	0.121	$1.17 \cdot 10^{-05}$	$4.84 \cdot 10^{-02}$	chr14:99736489	BCL11B	Body
cg19901403	0.02	0.241	$1.17 \cdot 10^{-05}$	$4.84 \cdot 10^{-02}$	chr8:74332522		
cg12618546	0.001	0.017	$1.19 \cdot 10^{-05}$	$4.86 \cdot 10^{-02}$	chr1:32817463		
cg20739354	0.039	0.834	$1.24 \cdot 10^{-05}$	$4.90 \cdot 10^{-02}$	chr1:153898552		
cg13996405	0.048	0.498	$1.24 \cdot 10^{-05}$	$4.90 \cdot 10^{-02}$	chr13:36920771	SPART	TSS200/5'UTR/1stExon
cg16551240	-0.068	0.619	$1.22 \cdot 10^{-05}$	$4.90 \cdot 10^{-02}$	chr10:134690534		
cg24737761	-0.064	0.644	$1.28 \cdot 10^{-05}$	$4.94 \cdot 10^{-02}$	chr6:106245659		
cg17748900	0.014	0.822	$1.28 \cdot 10^{-05}$	$4.94 \cdot 10^{-02}$	chr1:2041764	PRKCZ	5'UTR/Body
cg06133097	-0.01	0.92	$1.33 \cdot 10^{-05}$	$4.94 \cdot 10^{-02}$	chr7:32552212	AVL9	Body
cg10249246	0.047	0.415	$1.34 \cdot 10^{-05}$	$4.94 \cdot 10^{-02}$	chr14:105877289		
cg13264341	0.005	0.04	$1.35 \cdot 10^{-05}$	$4.94 \cdot 10^{-02}$	chr2:118572290	DDX18	5'UTR/1stExon
cg12501888	0.005	0.037	$1.38 \cdot 10^{-05}$	$4.94 \cdot 10^{-02}$	chr15:85177176	SCAND2P	Body
cg07163456	-0.074	0.44	$1.37 \cdot 10^{-05}$	$4.94 \cdot 10^{-02}$	chr3:192636424	MB21D2	TSS1500
**cg19872068**	**-0.012**	**0.863**	**$1.36 \cdot 10^{-05}$**	**$4.94 \cdot 10^{-02}$**	**chr17:44250734**	**KANSL1**	**TSS1500**
cg01102073	0.043	0.702	$1.38 \cdot 10^{-05}$	$4.94 \cdot 10^{-02}$	chr3:128204813	GATA2	Body

Supplementary Table 17: DMRs detected with bumphunter

Chr	Start	End	Value	Area	P Value	FWER	P Value Area	FWER Area
17	43663579	43663579	0.38	0.38	$<10^{-16}$	$<10^{-16}$	0.01	0.05
17	44343683	44343683	-0.17	0.17	$2.46 \cdot 10^{-04}$	$1.00 \cdot 10^{-03}$	0.12	0.33

Supplementary Table 18: DMRs detected with DMRcate

Chr	Start	End	N CpGs	minfdr	Stouffer	maxbetafc	meanbetafc
17	43662623	43663579	4	$3.18 \cdot 10^{-210}$	$7.24 \cdot 10^{-26}$	0.38	0.10
17	44343683	44343776	2	$1.91 \cdot 10^{-18}$	$2.04 \cdot 10^{-10}$	-0.17	-0.12

Supplementary Table 19: Cox regression models between CpGs and colorectal prognosis. In bold, variables significantly associated to disease-free survival.

Univariate

	cg08283464		cg03999934	
	HR (95% CI)	P value	HR (95% CI)	P value
CpG	**0.06 (0.006-0.52)**	**0.01**	**0.46 (0.25-0.84)**	**$1.1 \cdot 10^{-2}$**

Multivariate

	cg08283464		cg03999934	
	HR (95% CI)	P value	HR (95% CI)	P value
CpG	**0.02 ($4.9 \cdot 10^{-3}$-0.47)**	**0.017**	**0.03 (0.005-0.25)**	**$9.9 \cdot 10^{-4}$**
Age	1.00 (0.98-1.04)	0.47	1.01 (0.98-1.03)	0.65
Sex	0.81 (0.45-1.44)	0.47	0.93 (0.53-1.66)	0.82
Stage II	2.19 (0.63-7.57)	0.21	2.24 (0.65-7.73)	0.20
Stage III	2.91 (0.85-10.02)	0.09	3.19 (0.93-10.94)	0.07
Stage IV	**14.41 (4.04-51.4)**	**$3.9 \cdot 10^{-5}$**	**12.97 (3.64-46.20)**	**$7.7 \cdot 10^{-5}$**
PC1	3.68 (0.38-35.87)	0.26	3.37 (0.33-33.98)	0.30
PC2	1.01 (0.11-9.5)	0.99	0.73 (0.08-7.01)	0.79
PC3	1.41 (0.38-5.15)	0.61	1.16 (0.35-3.85)	0.81
PC4	6.37 (0.91-49.80)	0.06	3.12 (0.69-14.22)	0.14
